# The impact of high-risk lifestyle factors on all-cause mortality in the US non-communicable disease population

**DOI:** 10.1186/s12889-023-15319-1

**Published:** 2023-03-02

**Authors:** Ying Li, Xue Fan, Lifeng Wei, Kai Yang, Mingli Jiao

**Affiliations:** 1 Department of Science and Education, the Third People’s Hospital of Longgang District Shenzhen, Shenzhen, 518100 China; 2 The Personnel Department, Harbin Medical University, Harbin, 150086 China; 3 Shenzhen Institute of Respiratory Diseases, Shenzhen People’s Hospital (Second Clinical Medical College of Jinan University, First Affiliated Hospital of Southern University of Science and Technology), Shenzhen, 518001 China; 4 Research Center of Health Policy and Hospital Management, School of Health Management, Harbin Medical University, Harbin, 150086 China

**Keywords:** Lifestyle, Mortality, Non-communicable disease, Physical activity, Sedentary behavior, Dietary inflammatory index

## Abstract

**Background:**

Previous studies have suggested that lifestyle factors are associated with mortality in different population. However, little is known about the impact of lifestyle factors on all-cause mortality in non-communicable disease (NCD) population.

**Methods:**

This study included 10,111 NCD patients from the National Health Interview Survey. The potential high-risk lifestyle factors were defined as smoking, excessive drinking, abnormal body mass index, abnormal sleep duration, insufficient physical activity (PA), overlong sedentary behavior (SB), high dietary inflammatory index (DII) and low diet quality. Cox proportional hazard model was used to evaluate the impact of the lifestyle factors and the combination on all-cause mortality. The interaction effects and all combinations of lifestyle factors were also analyzed.

**Results:**

During 49,972 person-years of follow-up, 1040 deaths (10.3%) were identified. Among eight potential high-risk lifestyle factors, smoking (HR = 1.25, 95% CI 1.09–1.43), insufficient PA (HR = 1.86, 95% CI 1.61–2.14), overlong SB (HR = 1.33, 95% CI 1.17–1.51) and high DII (HR = 1.24, 95% CI 1.07–1.44) were risk factors for all-cause mortality in the multivariable Cox proportional regression. The risk of all-cause mortality was increased linearly as the high-risk lifestyle score increased (P for trend < 0.01). The interaction analysis showed that lifestyle had stronger impact on all-cause mortality among patients with higher education and income level. The combinations of lifestyle factors involving insufficient PA and overlong SB had stronger associations with all-cause mortality than those with same number of factors.

**Conclusion:**

Smoking, PA, SB, DII and their combination had significant impact on all-cause mortality of NCD patients. The synergistic effects of these factors were observed, suggesting some combinations of high-risk lifestyle factor may be more harmful than others.

**Supplementary Information:**

The online version contains supplementary material available at 10.1186/s12889-023-15319-1.

## Background

Non-communicable diseases (NCDs), primarily including cardiovascular disease (CVD), diabetes, chronic respiratory disease (CRD) and cancers, are the leading causes of premature death worldwide, and responsible for more than one-half of global disease burden and 70% of global deaths [1, 2]. Unhealthy lifestyle behaviors, such as smoking, drinking, insufficient physical activity and poor dietary pattern, are important risk factors of NCDs [3, 4]. Previous studies have also proved the impact of lifestyle on mortality of different populations [5–9]. Hence, a comprehensive understanding of these modifiable lifestyle factors on all-cause mortality of NCD patients would be beneficial for the prevention of premature death due to these factors.

Smoking, drinking, body mass index (BMI), physical activity (PA), diet quality and their combinations were the key lifestyle factors in previous studies [6, 10–12]. Recent studies in Denmark, Spain and China reported that healthy lifestyle behaviors could lower the risk of mortality from all cause, CVD and cancer [6–8]. An earlier meta-analysis also showed that the combination of healthy lifestyle factors could lowered the all-cause mortality risk by 66% [13]. However, few studies have focused on the impact of lifestyle factors on all-cause mortality in NCD patients, which deserved further attention. Moreover, some emerging and modern lifestyle factors may also have some influence on mortality risk [9, 14, 15].

In the current study, using a nationally representative population of NCD patients in the United States, we aimed to assess the impact of eight lifestyle factors on all-cause mortality of NCD patients. To assess whether the impacts differed in specific patient groups, we investigated the interaction effects between lifestyle factors and other variables. The mutually combinations of selected high-risk lifestyle factors were also created to explore the prevalence of these combinations and risk of mortality.

## Methods

### Study population

**Fig. 1:**
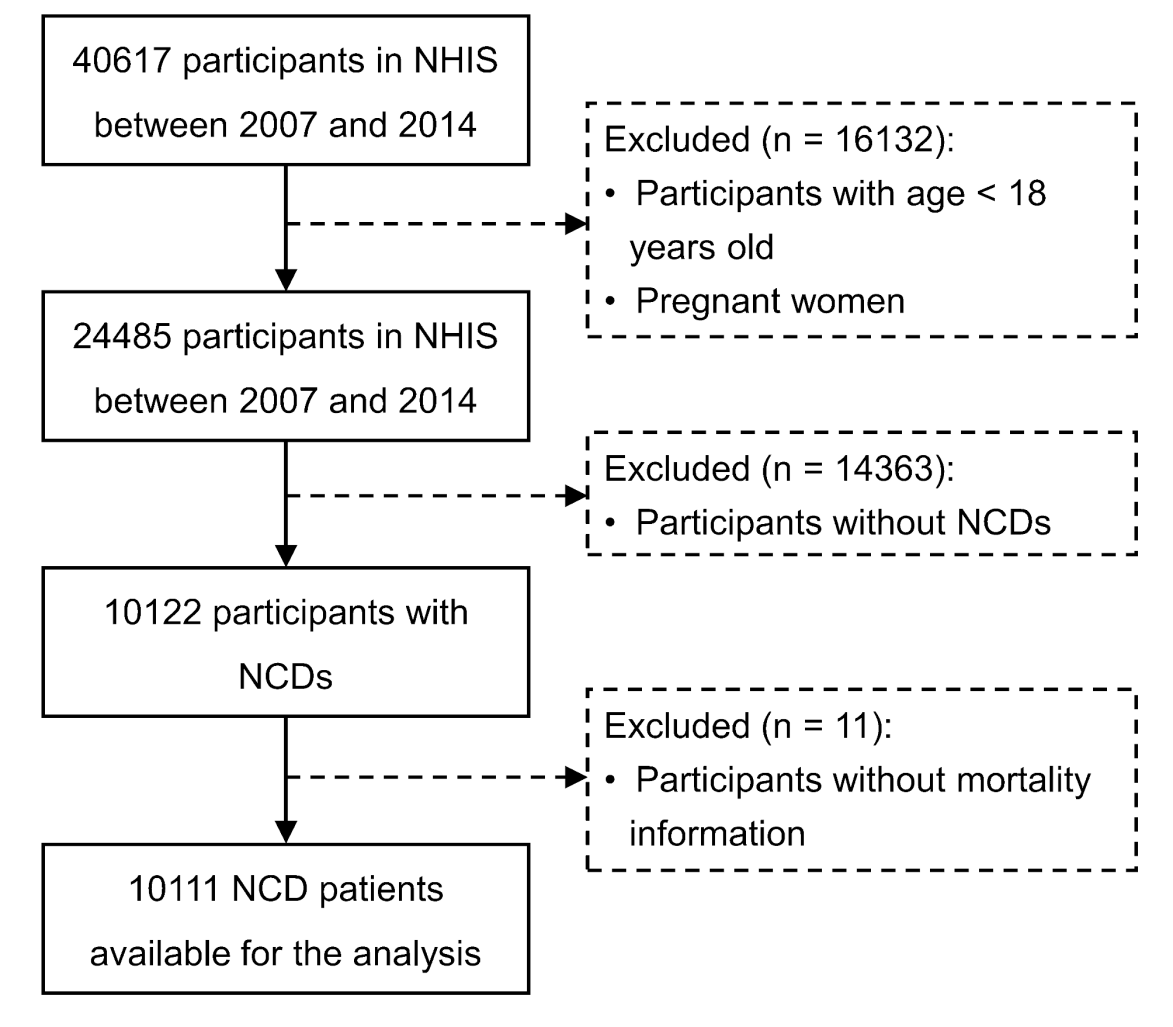
Flow diagram of participants in the study

This study included participants ≥ 18 years old from the National Health Interview Survey (NHIS) during 2007–2014, which was an ongoing national cross-sectional survey administered by the National Center for Health Statistics and the Centers for Disease Control and Prevention. The matched mortality information for these participants were derived from the National Death Index by 31 December 2015. Participants were also excluded because of pregnancy, without NCDs, or missing data on mortality information. Finally, a total of 10,111 NCD patients were included in this study (Fig. 1). The NCDs mainly included metabolic syndromes (MS), CVD, CRD and cancer in this study (Supplementary Methods).

### Assessment of lifestyle factors

Lifestyle factors included in the current study were BMI, smoking, drinking, sleep duration, PA, sedentary behavior (SB), inflammatory potential of diet measured by dietary inflammatory index (DII) and diet quality measured by healthy eating index (HEI) 2015 [16, 17]. Potential high-risk lifestyle factors were defined as smoking, excessive drinking (> 30 g/day for males and > 15 g/day for females), BMI < 18.5 or > 24.9 kg/m^2^, sleep duration < 7 or > 9 h/day, PA < 150 min/week, SB > 5 h/day, DII in the top 60%, and HEI-2015 in the bottom 60% [18–20]. For each factor, the patients received 1 point if he/she met these criterions, otherwise they received 0 point for the factor. After the multivariable Cox proportional regression for these factors, the independent high-risk lifestyle factors positively related to mortality were selected to create high-risk lifestyle scores. Details of PA, DII and HEI-2015 were provided in the Supplementary Method and Supplementary Table 1.

### Covariates

Covariates were included in the multivariable models to reduce confounding: sex (male and female), age group (< 60 years old and 60 ~ years old), race/ethnicity (Mexican American, other Hispanic, non-Hispanic white, non-Hispanic black and other race), marital status (married/living with partner, and widowed/divorced/separated/never married), education level (middle school or less, high school or equivalent, and college or more), income level (low, middle and high), MS, CVD, CRD and cancer.

### Statistical analysis

Baseline characteristics were presented as numbers and percentages for categorical variables and means and standard deviations for continuous variables, and compared using chi-square test or analysis of variance as appropriate. Before the primary analyses, the variables with missing values were imputed using random forest imputation approach, which was available in “missForest” R package [21]. Random forest imputation was a nonparametric algorithm that involved a series of multiple imputations until convergence.

Cox proportional hazard model was used to calculate hazards radio (HR) and corresponding 95% confidence intervals (CI) to evaluate the impact of 8 lifestyle factors on all-cause mortality. The Kaplan-Meier survival curves with log-rank tests were used to compare the survival among different high-risk lifestyle scores, which was calculated from the independent high-risk lifestyle factors in the Cox model. The attributable risks for different number of high-risk lifestyles were calculated according to previous study [22].

To explore the possibility of interaction effects, the interactions between high-risk lifestyle scores and other covariates were included in the multivariable Cox models, respectively. We also made stratified analyses by the covariates whose interaction effects were statistically significant. To compare specific patterns of high-risk lifestyles, 15 possible mutually exclusive combinations of 4 lifestyle factors were created. The prevalence and HR (95% CI) for each combination were presented.

Potential confounding factors were adjusted in all multivariable Cox models, including sex, age, race/ethnicity, marital status, education level, income level, MS, CVD, CRD and cancer. The multi-categorical variables were transformed into dummy variables in the models. Variable assignments were listed in Supplementary Table 2. All statistical analyses were performed using SAS 9.4 (SAS Institute Inc, Cary, North Carolina), and a *P* value < 0.05 was considered statistically significant.

## Results

### Descriptive statistics

During 49,972 person-years of follow-up, 1040 deaths (10.3%) were identified among 10,111 NCD patients, which was increased with number of potential high-risk lifestyle factors (Supplementary Table 3). Patient characteristics were shown in Table 1. The average age of the study population was 55.0 years, 48.7% of the patients were male, 56.4% were married or living with partner, 45.2% had a high household income, 46.8% had an education level of college, and 29.7% had two or more NCDs. Among the lifestyle factors, 79.1% had abnormal BMI, 51.5% were smokers, 12.1% consumed excessive alcohol, 45.0% had abnormal sleep duration, 52.8% had insufficient PA, 51.0% had overlong SB, 60% had pro-inflammatory diet, and 60% had low diet quality. As the number of potential high-risk lifestyle factors increased, the distributions of sex, race/ethnicity, marital status, income level, education level and NCD prevalence have changed. More specifically, the proportions of male and married/living with partner, income level and education level were decreased, and NCD prevalence was increased.

**Table 1:** Patient characteristics at baseline according to the number of potential high-risk lifestyle factors

Characteristics	Number of potential high-risk lifestyle factors^*^					Total
	0 ~ 2	3	4	5	6 ~ 8	
** N, %**	1595(15.8)	1967(19.5)	2552(25.2)	2214(21.9)	1783(17.6)	10,111(100.0)
**Age, year**	54.8 ± 19.0	54.6 ± 18.1	54.5 ± 18.0	55.6 ± 17.1	55.7 ± 16.5	55.0 ± 17.7
**Male, %**	785(49.2)	1007(51.2)	1278(50.1)	1040(47.0)	812(45.5)	4922(48.7)
**Dead, %**	108(6.8)	141(7.2)	244(9.6)	280(12.7)	267(15.0)	1040(10.3)
**Race/ethnicity**						
Mexican American	215(13.5)	287(14.6)	346(13.6)	253(11.4)	148(8.3)	1249(12.4)
Other Hispanic	161(10.1)	201(10.2)	251(9.8)	184(8.3)	139(7.8)	936(9.3)
Non-Hispanic white	842(52.8)	947(48.1)	1215(47.6)	1113(50.3)	965(54.1)	5082(50.3)
Non-Hispanic black	216(13.5)	377(19.2)	591(23.2)	560(25.3)	462(25.9)	2206(21.8)
Other Race	161(10.1)	155(7.9)	149(5.8)	104(4.7)	69(3.9)	638(6.3)
**Married/living with partner, %**	963(60.4)	1140(58)	1445(56.6)	1215(54.9)	940(52.7)	5703(56.4)
**Income level**						
Low income	434(27.2)	618(31.4)	933(36.6)	854(38.6)	793(44.5)	3632(35.9)
Middle income	275(17.2)	397(20.2)	465(18.2)	440(19.9)	328(18.4)	1905(18.8)
High income	886(55.6)	952(48.4)	1154(45.2)	920(41.6)	662(37.1)	4574(45.2)
**Education level**						
< High school	321(20.1)	491(25.0)	745(29.2)	707(31.9)	578(32.4)	2842(28.1)
High school or equivalent	346(21.7)	481(24.5)	653(25.6)	577(26.1)	476(26.7)	2533(25.1)
> High school	928(58.2)	995(50.6)	1154(45.2)	930(42.0)	729(40.9)	4736(46.8)
**CRD, %**	833(52.2)	1010(51.4)	1272(49.8)	1118(50.5)	961(53.9)	5194(51.4)
**CVD, %**	256(16.1)	332(16.9)	468(18.3)	503(22.7)	479(26.9)	2038(20.2)
**MS, %**	546(34.2)	844(42.9)	1207(47.3)	1085(49.0)	967(54.2)	4649(46.0)
**Cancer, %**	383(24.0)	383(19.5)	474(18.6)	419(18.9)	345(19.4)	2004(19.8)
**Number of NCD**						
1	1242(77.9)	1468(74.6)	1850(72.5)	1489(67.3)	1061(59.5)	7110(70.3)
2	285(17.9)	403(20.5)	550(21.6)	555(25.1)	505(28.3)	2298(22.7)
3	66(4.1)	89(4.5)	137(5.4)	154(7.0)	187(10.5)	633(6.3)
4	2(0.1)	7(0.4)	15(0.6)	16(0.7)	30(1.7)	70(0.7)
**BMI, kg/m** ^**2**^						
< 18.5	7(0.4)	17(0.9)	32(1.3)	35(1.6)	44(2.5)	135(1.3)
18.5 ~ 25	714(44.8)	540(27.5)	468(18.3)	298(13.5)	96(5.4)	2116(20.9)
25 ~ 30	437(27.4)	618(31.4)	838(32.8)	721(32.6)	609(34.2)	3223(31.9)
30~	437(27.4)	792(40.3)	1214(47.6)	1160(52.4)	1034(58.0)	4637(45.9)
**Smoking, %**	339(21.3)	745(37.9)	1251(49.0)	1377(62.2)	1499(84.1)	5211(51.5)
**Excessive drinking, %**	88(5.5)	207(10.5)	279(10.9)	286(12.9)	367(20.6)	1227(12.1)
**Abnormal sleep duration, %**	260(16.3)	613(31.2)	1073(42.1)	1256(56.7)	1349(75.7)	4551(45.0)
**Insufficient PA, %**	218(13.7)	568(28.9)	1134(44.4)	1360(61.4)	1492(83.7)	5339(52.8)
**Overlong SB, %**	361(22.6)	718(36.5)	1188(46.6)	1375(62.1)	1510(84.7)	5152(51.0)
**High diet inflammation, %**	286(17.9)	813(41.3)	1610(63.1)	1741(78.6)	1617(90.7)	6067(60.0)
**Low diet quality, %**	247(15.5)	810(41.2)	1589(62.3)	1759(79.5)	1662(93.2)	6067(60.0)

^*^The differences across the five groups were statistically significant except for age and CRD.

### Lifestyle factors and all-cause mortality

When eight individual lifestyle factors were entered in the model with other covariates, five were independently associated with all-cause mortality (Table 2). Of them, the increase of BMI had significantly decreased HR for all-cause mortality. Smoking (HR = 1.25, 95% CI 1.09–1.43), insufficient PA (HR = 1.86, 95% CI 1.61–2.14), overlong SB (HR = 1.33, 95% CI 1.17–1.51) and high DII (HR = 1.24, 95% CI 1.07–1.44) were risk factors for all-cause mortality. However, drinking, sleep duration and HEI-2015 showed no association with all-cause mortality.

**Table 2:** Multivariable Cox proportional regression for the impact of 8 lifestyle factors on all-cause mortality

Variables	*β*	*P*	*HR* (95% CI)
**BMI**			
< 18.5	0.24	0.28	1.27(0.82, 1.95)
18.5 ~ 25	-	-	1.00(Ref.)
25 ~ 30	-0.21	0.01	0.81(0.68, 0.95)
30~	-0.39	< 0.01	0.67(0.57, 0.81)
**Smoking**	0.22	< 0.01	1.25(1.09, 1.43)
**Drinking**	-0.06	0.60	0.94(0.75, 1.18)
**Sleep duration**	0.08	0.19	1.09(0.96, 1.23)
**PA**	0.62	< 0.01	1.86(1.61, 2.14)
**SB**	0.28	< 0.01	1.33(1.17, 1.51)
**DII**	0.21	< 0.01	1.24(1.07, 1.44)
**HEI-2015**	-0.14	0.05	0.87(0.76, 1.00)
**Sex**	-0.41	< 0.01	0.67(0.58, 0.76)
**Age**	0.06	< 0.01	1.06(1.06, 1.07)
**Race/ethnicity**			
Mexican American	-	-	1.00(Ref.)
Other Hispanic	0.00	1.00	1.00(0.73, 1.37)
Non-Hispanic White	0.17	0.15	1.19(0.94, 1.51)
Non-Hispanic Black	0.28	0.03	1.32(1.03, 1.70)
Other Race	0.22	0.24	1.24(0.86, 1.79)
**Marital status**	0.30	< 0.01	1.36(1.19, 1.55)
**Education level**	-0.05	0.17	0.95(0.88, 1.02)
**Income level**	-0.13	< 0.01	0.88(0.81, 0.95)
**MS**	0.04	0.59	1.04(0.90, 1.20)
**CVD**	0.43	< 0.01	1.54(1.35, 1.76)
**CRD**	-0.02	0.78	0.98(0.86, 1.12)
**Cancer**	0.24	< 0.01	1.28(1.11, 1.47)

**Fig. 2:**
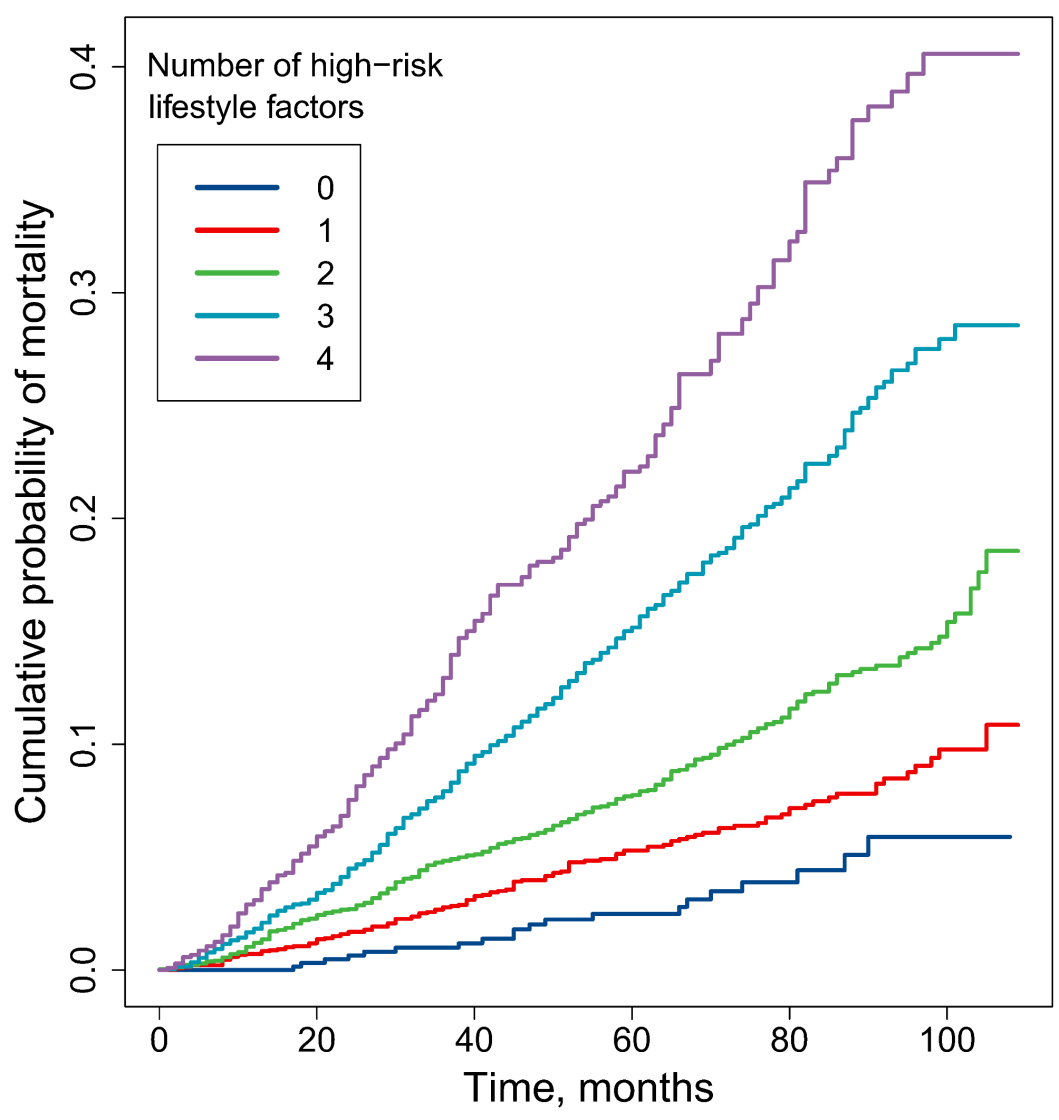
**Cumulative probability of mortality for NCD patients with different number of high-risk lifestyle factors.** The differences between any two survival curves were statistically significant (P < 0.05)

The risk of all-cause mortality was increased linearly as the high-risk lifestyle score increased, which was calculated from smoking, PA, SB and DII (P for trend < 0.01). Compared with patients without high-risk lifestyle, all-cause HRs for those with one, two, three, four high-risk lifestyle factors were 1.58, 2.14, 3.20 and 4.24, and the attributable risks were 2.43% 5.52% 11.59% and 17.69%, respectively (Table 3 and Supplementary Table 4). The cumulative mortality for patients with different high-risk lifestyle scores were statistically different (Fig. 2).

**Table 3:** Multivariable Cox proportional regression for the impact of high-risk lifestyle scores on all-cause mortality

Variables	*β*	*P*	*HR* (95% CI)
**High-risk lifestyle scores**			
0	-	-	1.00(Ref.)
1	0.46	0.06	1.58(0.98, 2.53)
2	0.76	< 0.01	2.14(1.36, 3.37)
3	1.16	< 0.01	3.20(2.03, 5.03)
4	1.45	< 0.01	4.24(2.66, 6.76)
**BMI**			
< 18.5	0.24	0.28	1.27(0.83, 1.95)
18.5 ~ 25	-	-	1.00(Ref.)
25 ~ 30	-0.21	0.01	0.81(0.69, 0.96)
30~	-0.38	< 0.01	0.68(0.57, 0.81)
**Drinking**	-0.08	0.51	0.93(0.74, 1.16)
**Sleep duration**	0.08	0.19	1.09(0.96, 1.23)
**HEI-2015**	-0.10	0.13	0.90(0.79, 1.03)
**Sex**	-0.36	< 0.01	0.70(0.61, 0.79)
**Age**	0.06	< 0.01	1.07(1.06, 1.07)
**Race/ethnicity**			
Mexican American	-	-	1.00(Ref.)
Other Hispanic	0.00	0.99	1.00(0.73, 1.37)
Non-Hispanic White	0.14	0.24	1.15(0.91, 1.45)
Non-Hispanic Black	0.27	0.03	1.31(1.02, 1.68)
Other Race	0.21	0.26	1.23(0.86, 1.77)
**Marital status**	0.30	< 0.01	1.36(1.19, 1.55)
**Education level**	-0.06	0.13	0.94(0.87, 1.02)
**Income level**	-0.14	< 0.01	0.87(0.81, 0.94)
**CRD**	-0.05	0.48	0.95(0.84, 1.09)
**CVD**	0.43	< 0.01	1.54(1.35, 1.76)
**MS**	0.04	0.55	1.04(0.91, 1.20)
**Cancer**	0.24	< 0.01	1.27(1.10, 1.46)

There were statistically significant heterogeneities regarding associations between high-risk lifestyle scores and all-cause mortality among some patient groups. The interactions between high-risk lifestyle scores, education level and income level were statistically significant (P < 0.01) (Supplementary Table 5). Stratified analysis suggested that high-risk lifestyle had stronger impact on all-cause mortality among patients with higher education level and higher income level (Supplementary Fig. 1). The association between high-risk lifestyle scores and all-cause mortality did not vary significantly by other patient groups.

### Combinations of high-risk lifestyle factors

All 15 mutually exclusive combinations of four high-risk lifestyle factors were presented in Table 4. Of these, 93.5% patients had one or more high-risk lifestyle factors, while only 6.5% had no high-risk lifestyle factors. Out of four single high-risk lifestyle factors, insufficient PA (HR = 2.14, 95% CI 1.20–3.81) had the strongest association with all-cause mortality, followed by smoking (HR = 1.93, 95% CI 1.16–3.23). Among the patients with two and three high-risk lifestyle factors, Insufficient PA + overlong SB (HR = 2.99, 95% CI 1.79-5.00) and Insufficient PA + overlong SB + High DII (HR = 3.66, 95% CI 2.27–5.91) had the greatest impact on the increased all-cause mortality, respectively.

**Table 4:** Prevalence of all combinations of 4 high-risk lifestyle factors and adjusted HR for their associations with all-cause mortality

Combinations of high-risk lifestyle factors	N (percent)	HR (95% CI)
No high-risk lifestyle factor	657(6.50)	1.00(Ref.)
Smoking	696(6.88)	1.93(1.16, 3.23)
Insufficient PA	319(3.15)	2.14(1.20, 3.81)
Overlong SB	572(5.66)	0.72(0.34, 1.54)
High DII	810(8.01)	1.35(0.79, 2.33)
Smoking + Insufficient PA	283(2.80)	2.24(1.28, 3.90)
Smoking + Overlong SB	516(5.10)	1.10(0.60, 2.02)
Smoking + High DII	911(9.01)	2.00(1.21, 3.29)
Insufficient PA + Overlong SB	470(4.65)	2.99(1.79, 5.00)
Insufficient PA + High DII	634(6.27)	2.40(1.45, 3.96)
Overlong SB + High DII	599(5.92)	2.08(1.18, 3.68)
Smoking + Insufficient PA + Overlong SB	531(5.25)	3.90(2.40, 6.33)
Smoking + Insufficient PA + High DII	649(6.42)	2.83(1.74, 4.59)
Smoking + Overlong SB + High DII	578(5.72)	2.21(1.30, 3.75)
Insufficient PA + Overlong SB + High DII	839(8.30)	3.66(2.27, 5.91)
Smoking + Insufficient PA + Overlong SB + High DII	1047(10.36)	4.21(2.64, 6.71)

## Discussion

In this study, we found that smoking, insufficient PA, overlong SB and high DII were independently associated with higher risk of all-cause mortality in NCD patients. The combined high-risk lifestyle scores, composite measures of these four factors, were significantly associated with increasing mortality as the number of high-risk factors increased. Stronger associations were observed among patients with higher education level and higher income level. The combinations involving insufficient PA and overlong SB had stronger associations with all-cause mortality than those with same number of factors.

Similar to some previous studies, this study found that smoking, insufficient PA and overlong SB were independently associated with higher all-cause mortality [6, 9]. Smoking was a well-established risk factor for some leading causes of death worldwide, such as CVD, lung cancer, lower respiratory infections and chronic obstructive pulmonary disease [23]. Insufficient PA was responsible for 9% of premature death of major NCDs [24]. The 2018 PA Guidelines for American recommended at least 75 min/week of vigorous-intensity PA or 150 min/week of moderate-intensity PA for substantial health benefit [25]. This study showed that even low-dose of PA can significantly reduce the all-cause mortality of NCD patients compared with physical inactivity. Besides displacing physical activity, SB and PA had independent effects on all-cause mortality, which meant that there were differences in the mechanisms of death caused by SB and PA.

Unhealthy diet was also a major risk factor for NCD globally, such as MS, CVD and certain types of cancer [26, 27]. The proportion of specific food elements (e.g., fruit, vegetable, milk) and some diet scores (e.g., HEI-2015, Mediterranean Diet) were applied in various studies to evaluate the diet pattern of lifestyle behaviors [8, 9, 19]. Compared with specific food elements, the comprehensive diet scores could better evaluate the effects of diet on health in its entirety. In previous studies on lifestyle, DII, which could characterize the inflammatory potential of diet, have not been included as a lifestyle factor. In the present study, DII (HR = 1.24, 95% CI 1.07–1.44) was a better index to evaluate the impact of diet on all-cause mortality than HEI-2015 (HR = 0.87, 95% CI 0.76-1.00).

Abnormal BMI and excessive drinking were not risk factors for all-cause mortality of NCD patients. The association of BMI with mortality was still controversial. A 2013 meta-analysis of 97 studies suggested that overweight might be protective factor for all-cause mortality [28]. U-shaped or J-shaped associations between BMI and all-cancer mortality have also been reported in previous studies [29, 30]. In this study, the increase of BMI was related with significantly lower all-cause mortality. The association between drinking and mortality risk also remained inconsistent. The cohort studies from Europe and the United States reported that excessive drinking was risk-factor for mortality, while moderate drinking is a protective factor [31, 32]. However, other studies showed that the risk of all-cause mortality rose with the level of drinking [33, 34]. Therefore, their effect on all-cause mortality still needed to be further verified by more sophisticated research.

Compared to most previous studies, we analyzed all the combinations of high-risk lifestyle factors, which showed that the same number of lifestyle factors contributed to mortality differently and that the combined effects may not be additive. The high-risk lifestyle factors tended to cluster, and 69.8% of patients had two or more high-risk lifestyle factors. Another interesting phenomenon was that the joint risk could be much higher than the sum of the individual risks. For example, although overlong SB on its own was not significantly associated with higher mortality risk, it augmented the risk noticeably when paired with insufficient PA and/or high DII (Table 4). This might indicate that overlong SB tended to be particularly harmful among those with insufficient PA and/or high DII. These findings suggested that there may be synergistic effects among lifestyle factors and that the epidemiological research and behavioral interventions should take into account the patterns of high-risk lifestyle and the interaction effects on all-cause mortality.

Our study has several limitations. First, most of the lifestyle factors were self-reported and thus some measurement errors were inevitable. However, the questionnaires for these factors have been validated and shown good reliability. Second, there were differences in the evaluation criteria of high-risk lifestyle factors, which might cause differences between studies. For example, the population of excessive drinking evaluated by drinking volume, frequency and pattern was likely to be different. Thresholds for sleep duration, SB, DII and HEI-2015 were also not defined. Third, the lifestyle information and covariates were collected at baseline. However, NCD patients were more likely to improve health status by changing high-risk lifestyle behaviors, which could not be considered in this study.

## Conclusion

In conclusion, our findings suggested that smoking, PA, SB, DII and their combination had significant impact on all-cause mortality of NCD patients. The synergistic effects of these impacts were observed, especially for overlong SB. The education level and income level could impact the association between high-risk lifestyle score and all-cause mortality. Hence, these interaction effects should be considered in behavioral interventions for NCD patients.

## Electronic supplementary material

Below is the link to the electronic supplementary material.

Supplementary Material 1

## Data Availability

This data can be accessed using the following link: https://www.cdc.gov/nchs/nhanes/index.htm.

## List of abbreviations

BMI: body mass index
CI: confidence intervals
CRD: chronic respiratory disease
CVD: cardiovascular disease
DII: dietary inflammatory index
HEI: healthy eating index
HR: hazards radio
MS: metabolic syndromes
NCDs: non-communicable diseases
NHIS: National Health Interview Survey
PA: physical activity
SB: sedentary behavior
