# Supplementary material for: The impact of high-risk lifestyle factors on all-cause mortality in the US non-communicable disease population
Source: BMC Public Health. 2023 Mar 2;23:422. doi: 10.1186/s12889-023-15319-1 (PMC9979572; doi:10.1186/s12889-023-15319-1)
Supplement: Supplementary file 1 — Supplementary Material 1 [file 12889_2023_15319_MOESM1_ESM.docx]

**Supplementary Method**

**Definition of 4 non-communicable diseases**

Metabolic syndrome (MS) was defined as the presence of 3 or more of the following 5 criteria: (1) abdominal obesity: waist circumference ≥ 102 cm for non-Hispanic white men, ≥ 88 cm for non-Hispanic white women, ≥ 94 cm for men of other races, and ≥ 80 cm for women of other races; (2) triglyceridemia ≥ 150 mg/dL; (3) high-density lipoprotein cholesterol <40 mg/dL for men and < 50 mg/dL for women; (4) SBP ≥ 130 mmHg, DBP ≥ 85 mmHg, or hypertension; and (5) fasting plasma glucose ≥ 100 mg/dL or DM^1^. DM was defined as a fasting plasma glucose ≥ 126 mg/dL, a glycated haemoglobin ≥ 6.5%, 2-hour glucose of Oral Glucose Tolerance Test ≥ 200 mg/dL, a self-reported physician diagnosis of DM, a use of glucose-lowering medications, or a use of insulin injections^2^.

Cardiovascular disease (CVD) was diagnosis according to self-reported physician diagnosis of congestive heart failure, coronary heart disease, angina pectoris, heart attack or stroke.

Chronic respiratory diseases (CRD) included participants with pre-bronchodilator FEV_1_/FVC < 0.7 or self-reported physician diagnosis of asthma, emphysema or chronic bronchitis.

The cancer included any cancer from blood, bone, brain, breast, cervix (cervical), colon, esophagus (esophageal), gallbladder, kidney, larynx/ windpipe, leukemia, liver, lung, lymphoma/hodgkin's disease, melanoma, mouth/tongue/lip, nervous system, ovary (ovarian), pancreas (pancreatic), prostate, rectum (rectal), skin (non-melanoma), skin (don't know what kind), soft tissue (muscle or fat), stomach, testis (testicular), thyroid, and uterus (uterine).

**Physical activity**

The physical activity (PA) questionnaire for participants was based on the Global Physical Activity Questionnaire (GPAQ) and includes questions related to occupation-related PA, leisure-time PA and transportation-related PA^3,4^. The intensity (vigorous versus moderate), frequency (per week), and duration (minutes) was evaluated for each type of PA in a typical week, in which one minute of vigorous-intensity PA is equivalent to two minutes of moderate-intensity PA. The occupation-related PA, leisure-time PA and transportation-related PA were summed to obtain the total amount of PA. According to the 2018 PA Guidelines for American, adults should be physically active with moderate-intensity for at least 150 to 300 minutes/week or with vigorous-intensity for 75 to 150 minutes/week or an equivalent combination of moderate- and vigorous-intensity PA for substantial health benefit^5^. Therefore, PA was categorized into two levels: insufficient PA (<150min/week) and sufficient PA (150min/week~).

**Dietary inflammatory index**

Evidences have indicated that diet played a central role in the regulation of chronic inflammation^6-8^. The dietary inflammatory index (DII) scores for participants were used to evaluate the inflammatory potential of diet, which were calculated based on the method previously described^9^. Briefly, *Z*-score and centered percentiles for each of the food parameters were derived from the intakes and reference global daily mean and standard deviation intake for these food parameters. The centered Z-scores were then multiplied by the food parameter-specific inflammatory effect score, which was derived and weighted according to 1943 articles. These values were summed to create the DII scores. In this study, 28 of the possible 45 food parameters were used to calculate the DII scores, including energy, carbohydrate, protein, total fat, saturated fat, monounsaturated fatty acids, polyunsaturated fatty acids, n-3 fatty acids, n-6 fatty acids, cholesterol, fiber, Vitamins (A, B1, B2, B3, B6, B12, C, D, E), folic acid, β-Carotene, Fe, Mg, Zn, Se, alcohol and caffeine. A higher DII score reflects stronger pro-inflammatory effect of the diet. No threshold has been defined for the DII score. Therefore, we selected participants with top 60% of DII as participants with high-risk lifestyle.

**Healthy eating index 2015**

The Healthy eating index (HEI) 2015 was used to evaluate diet quality of participants in this study, which can measure the compliance of diet to key recommendations in the 2015-2020 Dietary Guidelines for Americans^10^. The HEI-2015 consisted of 13 components, nine of which assessed adequacy (total fruit, whole fruit, total vegetables, greens and beans, whole grains, dairy, total protein foods, seafood and plant proteins, and Fatty Acids), and four of which assessed moderation (refined grains, sodium, percentage of energy from added sugars, and percentage of energy from saturated fatty acids). The consumption amounts of these components were standardized to 1000 calories basis, and points of 0-5 or 0-10 were assigned for each component according to Supplementary Table 1. The total HEI-2015 score ranged from 0 to 100, with higher scores being more compliant with the recommendations of diet guidelines as well as higher diet quality. No threshold has been defined for the HEI-2015. Therefore, we selected participants with bottom 60% of HEI-2015 as participants with high-risk lifestyle.

**References**

1. Alberti KG, Eckel RH, Grundy SM, et al. Harmonizing the metabolic syndrome: a joint interim statement of the International Diabetes Federation Task Force on Epidemiology and Prevention; National Heart, Lung, and Blood Institute; American Heart Association; World Heart Federation; International Atherosclerosis Society; and International Association for the Study of Obesity. *Circulation* 2009; **120**(16): 1640-5.

2. 2. Classification and Diagnosis of Diabetes: Standards of Medical Care in Diabetes-2018. *Diabetes Care* 2018; **41**(Suppl 1): S13-S27.

3. Bull FC, Maslin TS, Armstrong T. Global physical activity questionnaire (GPAQ): nine country reliability and validity study. *J Phys Act Health* 2009; **6**(6): 790-804.

4. Hallal PC, Andersen LB, Bull FC, Guthold R, Haskell W, Ekelund U. Global physical activity levels: surveillance progress, pitfalls, and prospects. *Lancet* 2012; **380**(9838): 247-57.

5. Piercy KL, Troiano RP, Ballard RM, et al. The Physical Activity Guidelines for Americans. *JAMA* 2018; **320**(19): 2020-8.

6. Giugliano D, Ceriello A, Esposito K. The effects of diet on inflammation: emphasis on the metabolic syndrome. *J Am Coll Cardiol* 2006; **48**(4): 677-85.

7. Galland L. Diet and inflammation. *Nutr Clin Pract* 2010; **25**(6): 634-40.

8. Christ A, Lauterbach M, Latz E. Western Diet and the Immune System: An Inflammatory Connection. *Immunity* 2019; **51**(5): 794-811.

9. Shivappa N, Steck SE, Hurley TG, Hussey JR, Hébert JR. Designing and developing a literature-derived, population-based dietary inflammatory index. *Public Health Nutr* 2014; **17**(8): 1689-96.

10. Krebs-Smith SM, Pannucci TE, Subar AF, et al. Update of the Healthy Eating Index: HEI-2015. *J Acad Nutr Diet* 2018; **118**(9): 1591-602.

Supplementary Table 1. Healthy Eating Index-2015 components, point values, and standards for scoring.

| Component | Maximum points | Standard for maximum score | Standard for minimum score of zero |
| --- | --- | --- | --- |
| **Adequacy** |  |  |  |
| Total Fruits | 5 | ≥0.8 c equivalents/1000 kcal | No fruit |
| Whole Fruits | 5 | ≥0.4 c equivalents/1000 kcal | No whole fruit |
| Total Vegetables | 5 | ≥1.1 c equivalents/1000 kcal | No vegetables |
| Greens and Beans | 5 | ≥0.2 c equivalents/1000 kcal | No dark green vegetables or beans and peas |
| Whole Grains | 10 | ≥1.5 oz equivalents/1000 kcal | No whole grains |
| Dairy | 10 | ≥1.3 c equivalents/1000 kcal | No dairy |
| Total Protein Foods | 5 | ≥2.5 oz equivalents/1000 kcal | No protein foods |
| Seafood and Plant Proteins | 5 | ≥0.8 c equivalents/1000 kcal | No seafood or plant proteins |
| Fatty Acids | 10 | (PUFAs+MUFAs)/SFAs≥2.5 | (PUFAs+MUFAs)/SFAs≤1.2 |
| **Moderation** |  |  |  |
| Refined Grains | 10 | ≤1.8 oz equivalents/1000 kcal | ≥4.3 oz equivalents/1000 kcal |
| Sodium | 10 | ≤1.1 g/1000 kcal | ≥2.0 g/1000 kcal |
| Added Sugars | 10 | ≤6.5% of energy | ≥26% of energy |
| Saturated Fats | 10 | ≤8% of energy | ≥16% of energy |

Note: PUFAs, polyunsaturated fatty acids; MUFAs, monounsaturated fatty acids; SFAs, saturated fatty acids.

Supplementary Table 2. Variable assignment for multivariable Cox proportional regression models in this study.

| Variables | Assignment |
| --- | --- |
| BMI | 1 = “<18.5”, 2 = “18.5~25”, 3 = “25~30”, 4 = “30~” |
| Smoking | 0 = “no”, 1 = “yes” |
| Drinking | 0 = “≤ 30 g/day for males and ≤ 15 g/day for females”, 1 = “> 30 g/day for males and > 15 g/day for females” |
| Sleep duration | 0 = “7~9 hours/day”, 1 = “<7 or >9 hours/day” |
| Physical activity | 0 = “150min/week~”, 1 = “<150min/week” |
| Sedentary behavior | 0 = “<6h/day”, 1 = “6h/day~” |
| DII | 0 = “<4.54”, 1 = “4.54~” |
| HEI-2015 | 0 = “<51.39”, 1 = “51.39~” |
| Sex | 0 = “male”, 1 = “female” |
| Age | Continuous |
| Race/ethnicity | 1 = “Mexican American”, 2 = “other Hispanic”, 3 = “non-Hispanic white”, 4 = “non-Hispanic black”, 5 = “other race” |
| Marital status | 0 = “married/living with partner”, 1 = “widowed/divorced/separated/never married” |
| Education level | 1 = “< high school”, 2 = “high school or equivalent”, 3 = “> high school” |
| Income level | 1 = “low income”, 2 = “middle income”, 3 = “high income” |
| MS | 0 = “no”, 1 = “yes” |
| CVD | 0 = “no”, 1 = “yes” |
| CRD | 0 = “no”, 1 = “yes” |
| Cancer | 0 = “no”, 1 = “yes” |

Supplementary Table 3. Underlying cause of 1040 deaths in this study.

| Cause | Number | Percentage |
| --- | --- | --- |
| Diseases of heart | 244 | 23.46 |
| Malignant neoplasms | 262 | 25.19 |
| Chronic lower respiratory | 61 | 5.87 |
| Accidents | 25 | 2.40 |
| Cerebrovascular diseases | 46 | 4.42 |
| Alzheimer’s disease | 18 | 1.73 |
| Diabetes mellitus | 33 | 3.17 |
| Influenza and pneumonia | 22 | 2.12 |
| Nephritis, nephrotic syndrome and nephrosis | 23 | 2.21 |
| All other causes | 271 | 26.06 |
| Not available | 35 | 3.37 |

Supplementary Table 4. The attributable risk for different high-risk lifestyle scores.

| High-risk lifestyle scores | Number of deaths | mortality | attributable risk |
| --- | --- | --- | --- |
| 0 | 20 | 3.04% | Reference |
| 1 | 131 | 5.47% | 2.43% |
| 2 | 292 | 8.56% | 5.52% |
| 3 | 380 | 14.63% | 11.59% |
| 4 | 217 | 20.73% | 17.69% |

Supplementary Table 5. The interaction effects between high-risk lifestyle scores and other factors in Cox regression models.

| Interaction items | *β* | *P* |
| --- | --- | --- |
| High-risk lifestyle scores * BMI | -0.04 | 0.27 |
| High-risk lifestyle scores * Drinking | -0.05 | 0.66 |
| High-risk lifestyle scores * Sleep duration | 0.03 | 0.60 |
| High-risk lifestyle scores * HEI-2015 | 0.07 | 0.30 |
| High-risk lifestyle scores * Sex | 0.04 | 0.50 |
| High-risk lifestyle scores * Age | 0.03 | 0.72 |
| High-risk lifestyle scores * Race/ethnicity | 0.03 | 0.39 |
| High-risk lifestyle scores * Marital status | 0.03 | 0.61 |
| High-risk lifestyle scores * Education level | 0.12 | <0.01 |
| High-risk lifestyle scores * Income level | 0.08 | 0.02 |
| High-risk lifestyle scores * CRD | 0.09 | 0.15 |
| High-risk lifestyle scores * CVD | 0.07 | 0.30 |
| High-risk lifestyle scores * MS | -0.03 | 0.66 |
| High-risk lifestyle scores * Cancer | 0.01 | 0.84 |


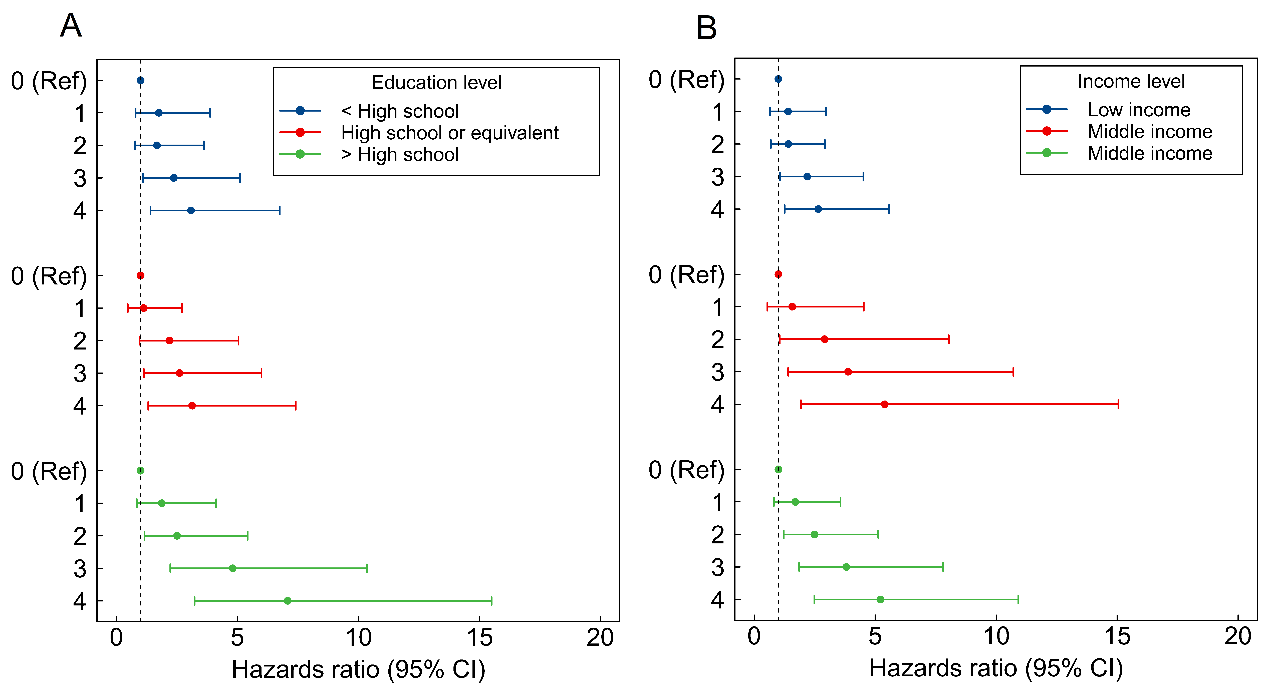


Supplementary Figure1. Subgroup analysis of the impact of lifestyle factors on all-cause mortality based on education level (A) and income level (B).
